# Case report: Strong low-density-cholesterol reduction accompanied by shrinkage of low-attenuation coronary plaque during lipid-lowering treatment with bempedoic acid—serial evaluation by coronary computed tomography angiography

**DOI:** 10.3389/fcvm.2023.1203832

**Published:** 2023-08-04

**Authors:** Grigorios Korosoglou, Alexander Giesen, Eva Geiss, Ksenija Stach

**Affiliations:** ^1^ Department of Cardiology, Vascular Medicine & Pneumology, GRN Hospital Weinheim, Weinheim, Germany; ^2^ Weinheim Imaging Center, Hector Foundation, Weinheim, Germany; ^3^ University of Heidelberg, Heidelberg, Germany; ^4^ Fifth Department of Medicine, University Medical Centre Mannheim, University of Heidelberg, Mannheim, Germany

**Keywords:** coronary artery disease, non-calcified plaque, hyperlipidemia, statin intolerance, bempedoic acid

## Abstract

Here, we present a patient with coronary artery disease and prior percutaneous coronary interventions. This patient had to discontinue taking multiple statins and ezetimibe due to intolerance with musculoskeletal complaints and nausea. Monotherapy with bempedoic acid was well tolerated and was exceptionally effective at lipid lowering, enabling patients to achieve the low-density lipoprotein target of <55 mg/dl, as recommended by current guidelines. In addition, serial coronary computed tomography angiography performed upon clinical indications, during 20 months of lipid-lowering treatment with bempedoic acid, demonstrated signs of favorable plaque component modification, with shrinkage of the low-attenuation plaque component compared to baseline findings.

## Introduction

Lipid-lowering therapy constitutes the cornerstone for the treatment of atherosclerotic cardiovascular disease. In this regard, extensive clinical and experimental evidence showed that low-density lipoprotein (LDL) is pivotal in the process of atherogenesis within the arterial wall (1). Therefore, current guidelines recommend a reduction of LDL-cholesterol of >50% from baseline and an LDL goal of <55 mg/dl in patients at very high risk for atherosclerotic cardiovascular disease both in primary and secondary prevention (2).

From a diagnostic point of view, coronary computed tomography angiography (CCTA) has emerged as the central non-invasive imaging technique not only for the detection of anatomically significant coronary artery disease (CAD) (3) but also for the evaluation of atherosclerotic plaque composition within the arterial wall (4, 5). In this regard, total low-attenuation plaque burden was reported as the most robust predictor of death and myocardial infarction, beyond stenosis severity in patients with CAD (4, 5). Statins, which are the primary lipid-lowering medications, were previously shown to enhance the progression of coronary artery calcification by repeated calcium-scoring scans (6). Recently, this effect was verified by serial CCTA scans, showing that statins result in slower plaque progression and transformation toward high-density calcium (7).

Recently, the non-statin lipid-lowering drug, bempedoic acid, was introduced and tested in high-risk patients under maximally tolerated statins and in statin-intolerant groups, exhibiting a good safety profile and effectiveness in terms of LDL reduction (8). However, data are scarce in terms of its potential effects on atherosclerotic plaque progression by serial CCTA studies.

## Case presentation

A 66-year-old female patient was initially referred to our outpatient center in March 2017 with suspected CAD due to exertional angina (CCS class II) and dyspnea. The patient had a history of arterial hypertension, which was treated with 5 mg ramipril per day, and hyperlipidemia, which was treated for 2 years with 10 mg simvastatin (initial LDL-cholesterol of 162 mg/dl). The ECG showed normal findings, and echocardiography revealed mild myocardial hypertrophy and normal ventricular diameters and function (LV ejection fraction of 62%).

Due to clinical symptoms and an intermediate pre-test probability of 16% (9), vasodilator stress cardiac magnetic resonance (CMR) was performed, the result of which exhibited myocardial perfusion abnormalities in the septal and inferior walls. No myocardial scars were detected through late gadolinium enhancement. Due to inducible myocardial ischemia in two myocardial segments, accompanied by persistent symptoms, coronary angiography was performed, confirming high-grade lesions in the left anterior descending artery (LAD) and right coronary artery (RCA). Percutaneous coronary intervention (PCI) was performed in both arteries, resulting in complete resolution of anginal symptoms thereafter.

**Figure 1:**
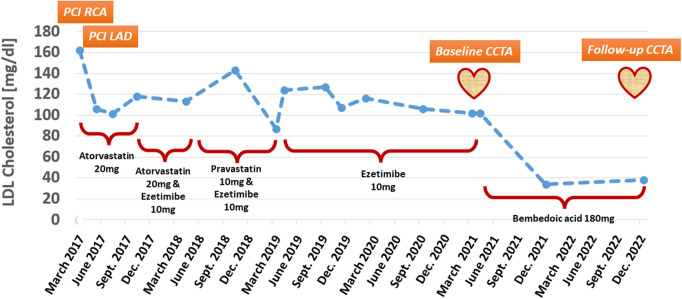
LDL-cholesterol during treatment with different lipid-lowering medications, including simvastatin, atorvastatin, pravastatin, ezetimibe, and bempedoic acid.

Lipid-lowering treatment was changed to 20 mg of atorvastatin per day, resulting in an LDL-cholesterol of 101 mg/dl in July 2017 and 118 mg/dl in October 2017. Since the target value of <70 mg/dl recommended by previous guidelines (10) could not be achieved, 10 mg of ezetimibe was added to the lipid-lowing medication, without significantly affecting LDL-cholesterol, which was measured at 113 mg/dl during April 2018. Figure 1 shows the serial LDL-cholesterol values between March 2017 and December 2022.

In June 2018, the patient started experiencing unsteadiness, weakness, hypoesthesia symptoms, and muscle pain in both legs. An electrophysiology examination of the peripheral muscles remained without detectable neurologic abnormalities, and statin-induced muscle symptoms (SAMS) were suspected. Atorvastatin (20 mg per day) was changed to pravastatin (10 mg per day) leading to clinical improvement of SAMS. However, LDL-cholesterol remained high, measuring 143 mg/dl in October 2018 (Figure 1).

In March 2019, the patient was referred to rheumatologists due to a recurrence of SAMS. Hereby, small fiber neuropathy (SFN) associated with anti-Mi-2 autoantibody-positive myositis was diagnosed. Since SFN encompasses several etiologies, such as diabetes, anti-retroviral, hypothyroidism, and hyperlipidemia, and has also been associated with statin therapy (11, 12), treatment with pravastatin was discontinued. SAMS further improved, and the patient was continued on treatment with 10 mg of ezetimibe per day. During this period from March 2019 to December 2020, LDL-cholesterol remained above the desired target value, ranging between 102 and 124 mg/dl (Figure 1).

**Figure 2:**
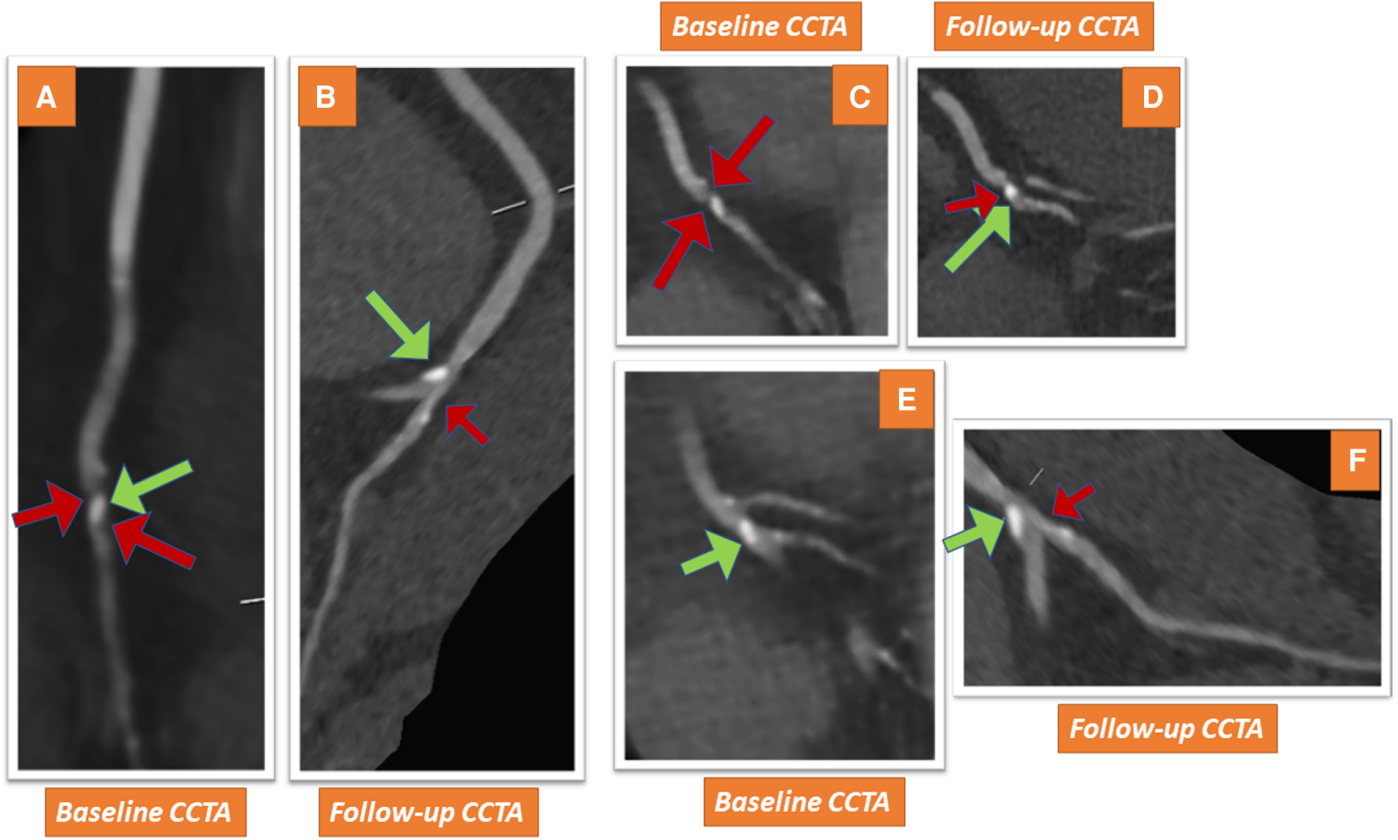
CCTA shows a non-calcified plaque and calcified components in the distal RCA of the patient (red and green arrows in **A–C**, pointing to low-attenuation and calcified plaque components, respectively). The lesion detected in the distal RCA reveals signs of favorable plaque component modification with follow-up CCTA after 20 months, exhibiting similar calcified plaque (green arrows in **D–F**), whereas low-attenuation plaque components are now hardly detectable (red arrows in **D–F**).

In March 2021, the patient reported new onset of atypical angina accompanied by exertional dyspnea. In addition, ezetimibe intolerance was suspected due to nausea. The ECG and echocardiography showed stable findings, and CCTA was performed to exclude the progression of CAD, including in-stent restenosis. CCTA was performed in a third-generation dual-source CT (SOMATOM Force, Siemens Healthineers, Forchheim, Germany), exhibiting patent stents in the LAD and RCA and any other high-grade stents. In the distal RCA, a moderate stenosis was detected, composed of non-calcified calcified components [Figures 2A, C, E, pointing to low-attenuation (red arrows) and calcified (green arrows) plaque components]. The plaque and both its calcified and non-calcified components were located directly at the crux of the RCA, resulting in a moderate ∼50%–70% diameter stenosis [coronary artery disease reporting and data system (CAD-RADS) 2.0 score of 3]. Repeated stress CMR showed normal perfusion, so invasive angiography was deferred. In addition, treatment with ezetimibe was discontinued due to recurrent episodes of nausea, and treatment with bempedoic acid was initiated. In November 2021, during a single treatment with bempedoic acid, the LDL-cholesterol was measured at 34 mg/dl and remained at 38 mg/dl on December 2022. Since the LDL target was now reached based on current guidelines (2), treatment with bempedoic acid was continued, and treatment with PSCK9 inhibitors was deferred.

In addition, a follow-up CCTA examination was performed on December 2022 (20 months after the initial CCTA) in our facility using the same third-generation dual-source CT scanner due to recurrent atypical symptoms. CCTA revealed patency of the stents in the LAD and the proximal RCA. Interestingly, the moderate lesion detected in the distal RCA in the baseline scan now revealed signs of plaque stabilization, exhibiting similar calcified plaque (green arrows in Figures 2B, D, F), whereas the low-attenuation plaque component was hardly detectable (small red arrows). In addition, the resultant lumen narrowing was now considered to be just mild, causing about 25%–50% diameter stenosis (CAD-RADS 2.0 score of 2). The patient was continued on treatment with 180 mg of bempedoic acid daily, and her subsequent clinical course in the next 4 months was uneventful.

## Discussion

This is to our knowledge the first case in the current literature, reporting on favorable plaque component modification by serial CCTA studies and shrinkage of low-attenuation plaque components in a patient, where lipid-lowering monotherapy with bempedoic acid achieved substantial LDL reduction, which could not be achieved by multiple statins and ezetimibe due to SAMS-associated intolerance effects.

Bempedoic acid, a non-statin lipid-lowering drug, which prevents cholesterol synthesis by inhibiting the action of the adenosine triphosphate (ATP) citrate lyase, a cytosolic enzyme upstream of 3-hydroxy-3-methylglutarylcoenzyme A reductase, is associated with a low incidence of muscle-related adverse events (13). In monotherapy of patients with statin intolerance, bempedoic acid reduces LDL levels by 23%, whereas LDL reduction may exceed about 40% in combination with ezetimibe (14). In addition, the recent CLEAR Outcomes study showed that treatment with bempedoic acid in statin-intolerant patients was associated with a lower risk of major adverse cardiovascular events, for the composite endpoint death from cardiovascular cause, non-fatal myocardial infarction, non-fatal stroke, and coronary revascularization (15). Previous studies reported the ability of bempedoic acid to reduce the high-sensitive C-reactive protein (hs-CRP) (8, 16), as a biomarker of low-grade inflammation, involved in atherosclerotic disease progression. This may add anti-inflammatory effects to the lipid-lowering potential of bempedoic acid.

Our case report highlights the potential of bempedoic acid for effective lipid lowering in a patient with statin and ezetimibe intolerance. In addition, its potential for the reduction of non-calcified burden components is described by serial CCTA images. It should be noted, however, that the patient had been on treatment with statins and ezetimibe for a considerable time before the initiation of treatment with bempedoic acid, which may have confounded the effects on plaque burden and composition, as described in this context. In addition, it needs to be noted that bempedoic acid was considered before the initiation of PCSK9 treatment due to the patient’s preference for oral over subcutaneous therapy and due to cost issues. Indeed, the current reimbursement system in Germany requires proof of administration of all possible sorts of lipid-lowing treatment, including statins, ezetimibe, and bempedoic acid prior to the approval of treatment with PCSK9 inhibitors. In our case, bempedoic acid reduced LDL-cholesterol by over 80% compared to the initial LDL values. Notably, relatively high inter-individual heterogeneity in LDL lowering from 0 to over 80% has been described in previous studies (17). Similar effects have previously been reported for statins, which may be the consequence of genetic polymorphisms modulating cholesterol homeostasis (18). Thus, our patient seems to be a hyper-responder to bempedoic acid in this context, where the substance achieved highly effective LDL reduction as well as favorable plaque modification. Future studies like the ongoing LOCATE trial (https://drks.de/search/de/trial/DRKS00031954) are now warranted to investigate the potential of bempedoic acid and other lipid-lowering or anti-inflammatory drugs on plaque modification within multi-center serial CCTA studies.

## Data Availability

The raw data supporting the conclusions of this article will be made available by the authors, without undue reservation.

## References

[1] Boren J Chapman MJ Krauss RM Packard CJ Bentzon JF Binder CJ Low-density lipoproteins cause atherosclerotic cardiovascular disease: pathophysiological, genetic, and therapeutic insights: a consensus statement from the European Atherosclerosis Society Consensus Panel. Eur Heart J. (2020) 41(24):2313–30. 10.1093/eurheartj/ehz962 32052833 PMC7308544

[2] Mach F Baigent C Catapano AL Koskinas KC Casula M Badimon L 2019 ESC/EAS guidelines for the management of dyslipidaemias: lipid modification to reduce cardiovascular risk. Eur Heart J. (2020) 41(1):111–88. 10.1093/eurheartj/ehz455 31504418

[3] Group DT Maurovich-Horvat P Bosserdt M Kofoed KF Rieckmann N Benedek T CT or invasive coronary angiography in stable chest pain. N Engl J Med. (2022) 386(17):1591–602. 10.1056/NEJMoa2200963 35240010

[4] Williams MC Kwiecinski J Doris M McElhinney P D’Souza MS Cadet S Low-attenuation noncalcified plaque on coronary computed tomography angiography predicts myocardial infarction: results from the multicenter SCOT-HEART trial (Scottish computed tomography of the HEART). Circulation. (2020) 141(18):1452–62. 10.1161/CIRCULATIONAHA.119.044720 32174130 PMC7195857

[5] Giusca S Schutz M Kronbach F Wolf D Nunninger P Korosoglou G. Coronary computer tomography angiography in 2021—acquisition protocols, tips and tricks and heading beyond the possible. Diagnostics. (2021) 11(6). 10.3390/diagnostics11061072 PMC8230532 34200866

[6] Dykun I Lehmann N Kalsch H Mohlenkamp S Moebus S Budde T Statin medication enhances progression of coronary artery calcification: the Heinz Nixdorf Recall Study. J Am Coll Cardiol. (2016) 68(19):2123–5. 10.1016/j.jacc.2016.08.040 27810054

[7] van Rosendael AR van den Hoogen IJ Gianni U Ma X Tantawy SW Bax AM Association of statin treatment with progression of coronary atherosclerotic plaque composition. JAMA Cardiol. (2021) 6(11):1257–66. 10.1001/jamacardio.2021.3055 34406326 PMC8374741

[8] Goit R Saddik SE Dawood SN Rabih AM Niaj A Raman A Bempedoic acid’s use as an adjunct in lowering low-density lipoprotein cholesterol in patients with coronary artery disease: a systematic review. Cureus. (2022) 14(10):e29891. 10.7759/cureus.29891 36348882 PMC9632934

[9] Knuuti J Wijns W Saraste A Capodanno D Barbato E Funck-Brentano C 2019 ESC guidelines for the diagnosis and management of chronic coronary syndromes. Eur Heart J. (2020) 41(3):407–77. 10.1093/eurheartj/ehz425 31504439

[10] Catapano AL Graham I De Backer G Wiklund O Chapman MJ Drexel H 2016 ESC/EAS guidelines for the management of dyslipidaemias. Eur Heart J. (2016) 37(39):2999–3058. 10.1093/eurheartj/ehw272 27567407

[11] Devigili G Tugnoli V Penza P Camozzi F Lombardi R Melli G The diagnostic criteria for small fibre neuropathy: from symptoms to neuropathology. Brain. (2008) 131(Pt 7):1912–25. 10.1093/brain/awn093 18524793 PMC2442424

[12] Lo YL Leoh TH Loh LM Tan CE. Statin therapy and small fibre neuropathy: a serial electrophysiological study. J Neurol Sci. (2003) 208(1–2):105–8. 10.1016/S0022-510X(02)00396-9 12639733

[13] Saeed A Ballantyne CM. Bempedoic acid (ETC-1002): a current review. Cardiol Clin. (2018) 36(2):257–64. 10.1016/j.ccl.2017.12.007 29609755

[14] Ray KK Bays HE Catapano AL Lalwani ND Bloedon LT Sterling LR Safety and efficacy of bempedoic acid to reduce LDL cholesterol. N Engl J Med. (2019) 380(11):1022–32. 10.1056/NEJMoa1803917 30865796

[15] Nissen SE Lincoff AM Brennan D Ray KK Mason D Kastelein JJP Bempedoic acid and cardiovascular outcomes in statin-intolerant patients. N Engl J Med. (2023) 388(15):1353–64. 10.1056/NEJMoa2215024 36876740

[16] Ridker PM Lei L Ray KK Ballantyne CM Bradwin G Rifai N. Effects of bempedoic acid on CRP, IL-6, fibrinogen and lipoprotein(a) in patients with residual inflammatory risk: a secondary analysis of the CLEAR harmony trial. J Clin Lipidol. (2023) 17(2):297–302. 10.1016/j.jacl.2023.02.002 36813656

[17] Warden BA Cardiology BA Purnell JQ Duell PB Fazio S. Real-world utilization of bempedoic acid in an academic preventive cardiology practice. J Clin Lipidol. (2022) 16(1):94–103. 10.1016/j.jacl.2021.11.013 34924351

[18] Karlson BW Wiklund O Palmer MK Nicholls SJ Lundman P Barter PJ. Variability of low-density lipoprotein cholesterol response with different doses of atorvastatin, rosuvastatin, and simvastatin: results from VOYAGER. Eur Heart J Cardiovasc Pharmacother. (2016) 2(4):212–7. 10.1093/ehjcvp/pvw006 27533947

